# A rare cause for intrathoracic mass in an elderly male

**DOI:** 10.4103/1817-1737.33705

**Published:** 2007

**Authors:** K. A. Vishak, K. B. Gireesh, Prasad Shiva, S. K. Jayarama

**Affiliations:** *Department of Tuberculosis and Chest Disease, Department of General Medicine and Radiotherapy, Kasturba Medical College Hospital, Mangalore, India*

An 86-year-old man, a known case of hypertension and chronic obstructive pulmonary disease (COPD), on inhalational steroids, presented to us with history of fever, cough and vomiting of 1-day duration. On physical examination, patient had a pulse of 90 beats per minute, blood pressure of 140/ 88 mmHg and respiratory rate of 18 per minute. Respiratory examination revealed the patient to have an emphysematous chest with bilateral diffuse expiratory rhonchi, basal crepitations and decreased breath sounds accompanied by a dull percussion note in left lower lobe area.

## Investigations

Routing blood investigations were normal except for mild leucocytosis. Chest Roentgenogram (PA view) showed a well-defined homogenous circular mass abutting lateral part of diaphragm and chest wall. In view of old age of the patient with risk factors including chronic smoking and COPD, a provisional diagnosis of bronchogenic carcinoma was made and a contrast-enhanced computerized tomographic (CECT) scan study was asked to confirm the diagnosis.

## Questions

- What radiological abnormalities are present on these images?
- What is the likely diagnosis in this case?

## Answers

**Figure 1:**
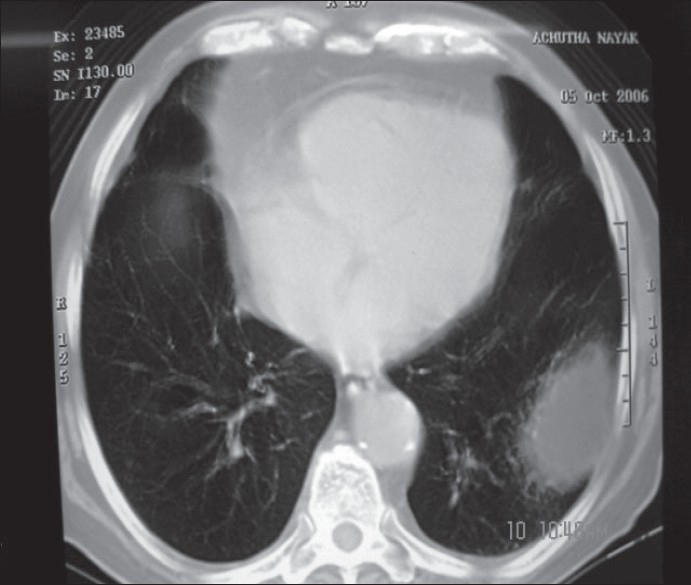
CECT scan showed a focal, well-defined nonenhancing hypodense area (-30 HU) in the posterior and lateral basal segments of left lower lobe, abutting lateral chest wall

**Figure 2:**
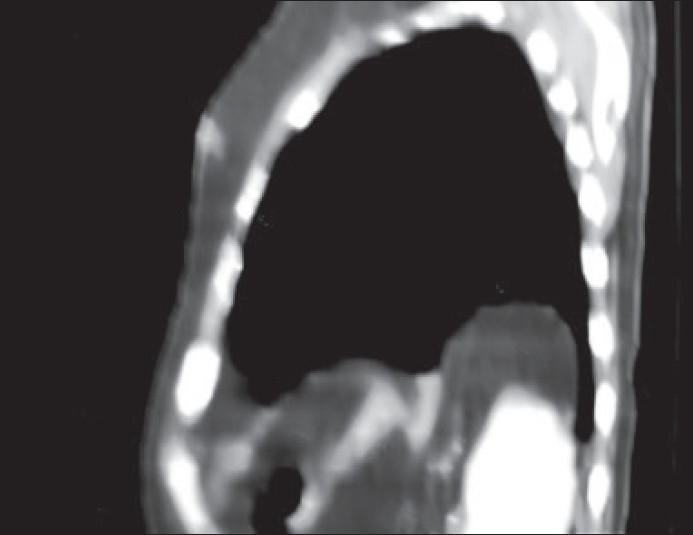
Reconstructed image in sagittal section shows the intrathoracic lesion to be continuous with greater omentum below
- Posterolateral diaphragmatic hernia - Bochdalek hernia

- CECT scan showed a focal, rounded well-defined non-enhancing hypodense area (-30 HU) measuring 8 × 8 × 4.2 × 5 cm in the posterior and lateral basal segments of left lower lobe, abutting lateral chest walls [Figure 1]. Since the lesion density was unusual for a mass lesion, the CECT scan images were reconstructed in the sagittal section [Figure 2], which showed the intrathoracic lesion to be continuous with greater omentum below.

## Discussion

Bochdalek hernia is a congenital defect of the posterolateral region of the diaphragm, usually on the left side, and is most often seen in infants but is rare in adults. Developmentally, diaphragm is a mesodermal partition originating mainly from septum transversum, which forms the central portion of it. The posterior part of the diaphragm develops from dorsal mesentery of the esophagus. On each side of the dorsal mesentery and behind the septum transversum, a triangular gap exists in early fetal life - known as pleuro-peritoneal opening or canal. Persistence of this canal leads to herniation of intra-abdominal organs into thoracic cavity. On account of this, Bochdalek hernia has no peritoneal sac. Rarely, it can also result due to a posttraumatic defect in the diaphragm.

Bochdalek hernias are uncommon and usually diagnosed on CT scan in about 5–10% of adults.[1,2] They are small in size, not well appreciated in routine chest radiographs, and are diagnosed only on CT scans as incidental findings. This incidence increases with age, suggesting that they are acquired. They are rare in patients younger than 40 years of age.[1]

Most of the adult cases are asymptomatic, and diagnosis is made incidentally during imaging. However if symptoms appear, they are generally non-acute and related to digestive tract, like dyspeptic symptoms, rarely necessitating emergency surgical treatment.[3,4] There are cases reported in which adult diaphragmatic hernias that are silent for many years, later growing rapidly and presenting with acute symptoms.[5]

It may be difficult or impossible to distinguish a posterolateral diaphragmatic defect from the much less frequent eventration of the diaphragm or an intrathoracic posteriorly situated lung mass, and these should be considered in the differential diagnosis of a posteriorly situated chest mass. Mortality rate is lower than 3% in adults, although it could rise until 32% if diagnosis and treatment are delayed.[6]

CT scan is the technique of choice for prompt and correct diagnosis. However, contrast imaging to demonstrate the density; and reconstruction of images in sagittal and coronal planes, as in our case, may be required to localize the intra-abdominal origin of these lesions. Upper gastrointestinal barium study may show the stomach inside the thoracic cavity. The unusual aspect of our case was the large size of Bochdalek hernia on presentation, which is quite uncommon as they are usually smaller-sized lesions diagnosed incidentally during CECT scans. We want to emphasize that late presentations of the lesion coupled with the intrathoracic location and risk factors masquerading as a neoplastic lung mass can be misleading even to an astute clinician.

In our case, the patient improved symptomatically with conservative management for chronic obstructive airway disease and was discharged from the hospital.
